# The toxic effects and possible mechanisms of Bisphenol A on oocyte maturation of porcine *in vitro*

**DOI:** 10.18632/oncotarget.8689

**Published:** 2016-04-11

**Authors:** Teng Wang, Jun Han, Xing Duan, Bo Xiong, Xiang-Shun Cui, Nam-Hyung Kim, Hong-Lin Liu, Shao-Chen Sun

**Affiliations:** ^1^ College of Animal Science and Technology, Nanjing Agricultural University, Nanjing 210095, China; ^2^ Department of Animal Sciences, Chungbuk National University, Cheongju 361-763, Korea

**Keywords:** BPA, cytoskeleton, autophagy/apoptosis, oxidative stress, epigenetics

## Abstract

Bisphenol A (BPA) and Di-(2-ethylhexyl) phthalate (DEHP) are widely used in the plastic industry such as water bottles, containers, packaging and toys. BPA and DEHP are shown to be the endocrine disruptors which disturb the endocrine system and are linked to several diseases including infertility. In this study, we investigated the effects of BPA exposure on porcine oocyte maturation and its possible reasons. Our results showed that: (i) the rates of oocyte maturation significantly decreased with 250 μM BPA treatment *in vitro*, but not DEHP. This might be due to the delayed cell cycle progression of oocyte maturation. (ii) BPA treatment resulted in abnormal cytoskeletons on porcine oocytes, showing with aberrant actin distribution, spindle morphology and chromosome alignment, which was further confirmed by the reduced p-MAPK level. (iii) The fluorescence intensity of histone methylation (H3K4me2) and DNA methylation (5 mC) levels were altered after BPA treatment, indicating that epigenetic modification was disturbed. (iv) BPA-exposed oocytes had higher rates of early stage apoptosis/autophagy, and this may be resulted from the increased level of oxidative stress. Collectively, our results indicated that porcine oocytes maturation was disrupted after BPA treatment through disrupting cytoskeletal dynamics, epigenetic modifications and inducing apoptosis/autophagy.

## INTRODUCTION

The growing evidence indicates that environmental contaminants can pose negatively risks to animals and human health. Because some of these substances can mimic and alter the actions of endogenous hormones, some potentially disrupting endocrine function in humans thereby they are referred to as endocrine disruptors (EDC) [1, 2]. Di(2-ethylhexyl) phthalate (diethyl-hexyl phthalate, DEHP) is widely used as a plasticizer in manufacturing products made by polyvinyl compounds, and it is an estrogen-like chemical [3, 4]. DEHP is reported as a potent reproductive toxicant and it can cause gonadal morphological or functional alterations in both sexes by acting as endocrine disruptors [5, 6]. Similarly to DEHP, Bisphenol A (BPA) is also a high production volume environmental estrogen-like chemical, and it is widely used in a variety of manufacturing polycarbonate plastic products posing a risk health to human [3]. In 2010, Canada is the first country that announced BPA is a toxic substance, and the European Union, Canada and the United States have banned BPA in baby bottles.

Some studies have shown that BPA could stimulate prolactin release [7, 8], alter thyroid hormone action [8, 9], impair aromatase expression [10] and act as an anti-androgen [11]. Human exposure to BPA is nearly ubiquitous and takes place through inhalation, ingestion, and dermal absorption. Females are born with a limited number of oocytes, it is significantly decreased in number from the second trimester of the fetal period until menopause through progressive apoptosis [12]. Recent studies have shown that BPA exposure resulted in developmental genitourinary anomalies, sperm abnormality and sperm DNA damage induced in spermatozoa and epigenetic modifications in off-spring, as well as decreased the epididymal weight and increased prostate weight in male rodents [13–16]. The toxic effects of BPA on oocyte quality have been shown with *in vitro* mouse models [17, 18], *in vivo* mice models [19–21], *in vitro* human models [22]. These previous studies have reported that BPA is toxic to fertilization, but most of these studies on oocytes maturation was from clinical aspect and the mechanisms of toxicity are still not fully understood. To find the causes and mechanism for toxic effects of BPA exposure on oocytes, we investigated this through epigenetic modification and apoptosis/autophagy aspects with the porcine model, since the genome of porcine is more close to human species, which could more concisely reflect the reproduction system of human.

Several cellular processes like epigenetic modifications, apoptosis/autophagy and oxidative stress are all critical for oocyte maturation. Apoptosis, a programmed cell death which includes prenatal germ cell death, granulosa cell death during post-natal follicular atresia, plays a major role in the elimination of germ cells at all the stages of oogenesis and ovulation [24, 25]. And autophagy influences maternal mRNA degradation and apoptosis during porcine parthenote development *in vitro* [26, 27]. Meanwhile, autophagy is also critical for in vitro maturation and improves the nuclear and cytoplasmic maturation of porcine oocytes [27]. Oxidative stress also inhibited oocyte maturation, and our previous study showed that HT-2 toxin induced oxidative stress inhibited mouse oocyte polar body extrusion [25]. Another report showed that oocytes maturation inhibited by oxidative stress could be protected from melatonin [28]. BPA was shown to induce oxidative stress which resulted in DNA damage in INS-1 cells [29].

Till now there is still little research focused on the influence on porcine oocyte maturation and especially the mechanisms of its toxicity. Therefore, the objective of the present study was to evaluate the influence of acute exposure to BPA and DEHP on porcine oocyte maturation, subcellular structure, epigenetic modification, oxidative stress, autophagy and apoptosis. Our study suggests that BPA disrupts porcine oocytes maturation through changing epigenetic modification, inducing oxidative stress, excessive autophagy and apoptosis.

## RESULTS

### BPA but not DEHP treatment results in the failure of polar body extrusion in porcine oocytes *in vitro*

**Figure 1:**
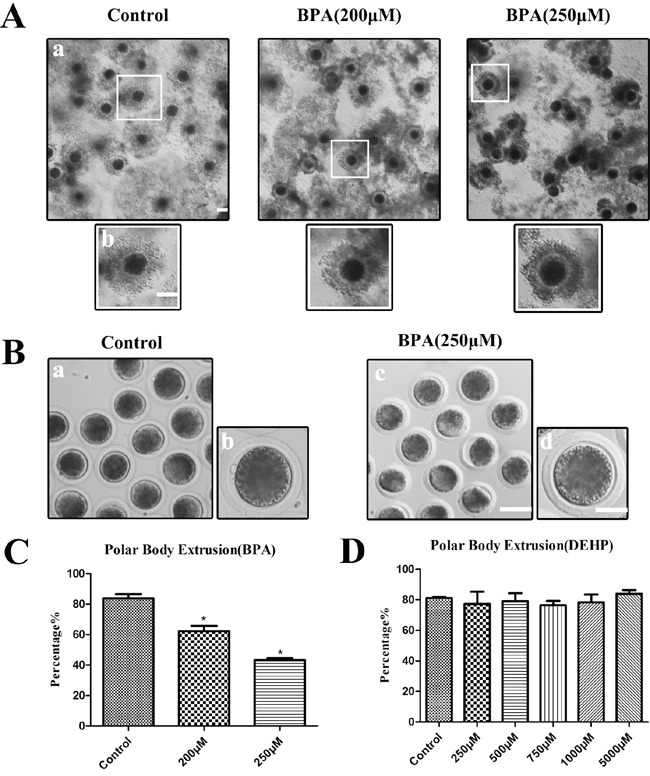
BPA treatment results in the failure of polar body extrusion in porcine oocytes but not DEHP in vitro **A.** Granule cell diffusion of COCs were weakened after BPA treatment. a, Bar = 100 μm; b, Bar = 50 μm. **B.** Oocytes failed to extrude polar bodies after BPA treatment. a, c Bar = 50 μm; b, d Bar = 20 μm. **C.** The polar body extrusion rate was significantly reduced after BPA treatment. Asterisks denote significantly different (P < 0.01). *significantly different (P < 0.01). **D.** The polar body extrusion rate no significantly variation after DEHP treatment (P > 0.05).

We first found that exposure to BPA had immediate impact on the expansion of intact porcine COC cultured *in vitro*. Porcine COCs were cultured in normal maturation medium for 44 h in the presence of BPA with different concentrations (200 μM, 250 μM) or DEHP (250 μM, 500 μM, 750 μM, 1 mM, 5 mM), and then the polar body extrusion rates were examined. As shown in Figure 1A, the good expansion of the peripheral layers of cumulus cells was observed in control maturation group, whereas it was much weaker in BPA treated COCs. Moreover, most of control oocytes had extruded polar bodies and were arrested at the MII stage, whereas in BPA treated oocytes polar body extrusion was suppressed (Figure 1B). BPA treatment resulted in reduced polar body extrusion in a dose-dependent manner (Figure 1C). BPA treatment effectively inhibited polar body emission at a concentration of 200 μM and 250 μM. These results showed that 83.67 ± 2.86% (n = 367 COCs) of control oocytes had extruded polar bodies. However, with 200 μM and 250 μM BPA treatment, the rates of polar body extrusion were significantly reduced to 62.32 ± 3.39% (P < 0.05; n = 165 COCs) and 43.19 ± 1.38% (P < 0.01; n = 146 COCs). The results suggest that exposure to BPA causes the failure of polar body extrusion in porcine oocytes. However, with 250 μM, 500 μM, 750 μM, 1 mM and 5 mM DEHP treatment, the rates of polar body extrusion had no significant variation, as shown in Figure 1D, the rates of polar body were: 81.18 ± 0.64% (n = 463) for controls vs. 79.16 ± 5.17% (n = 159) for 250 μM, 79.16 ± 5.17% (n = 177) for 500 μM, 76.39 ± 2.93% (n = 157) for 750 μM, 78.38 ± 5.10% (n = 145) for 1 mM, 84.05 ± 2.29% (n = 131) for 5 mM.

### Exposure to BPA disturbs the cell cycle of porcine oocyte maturation

**Figure 2:**
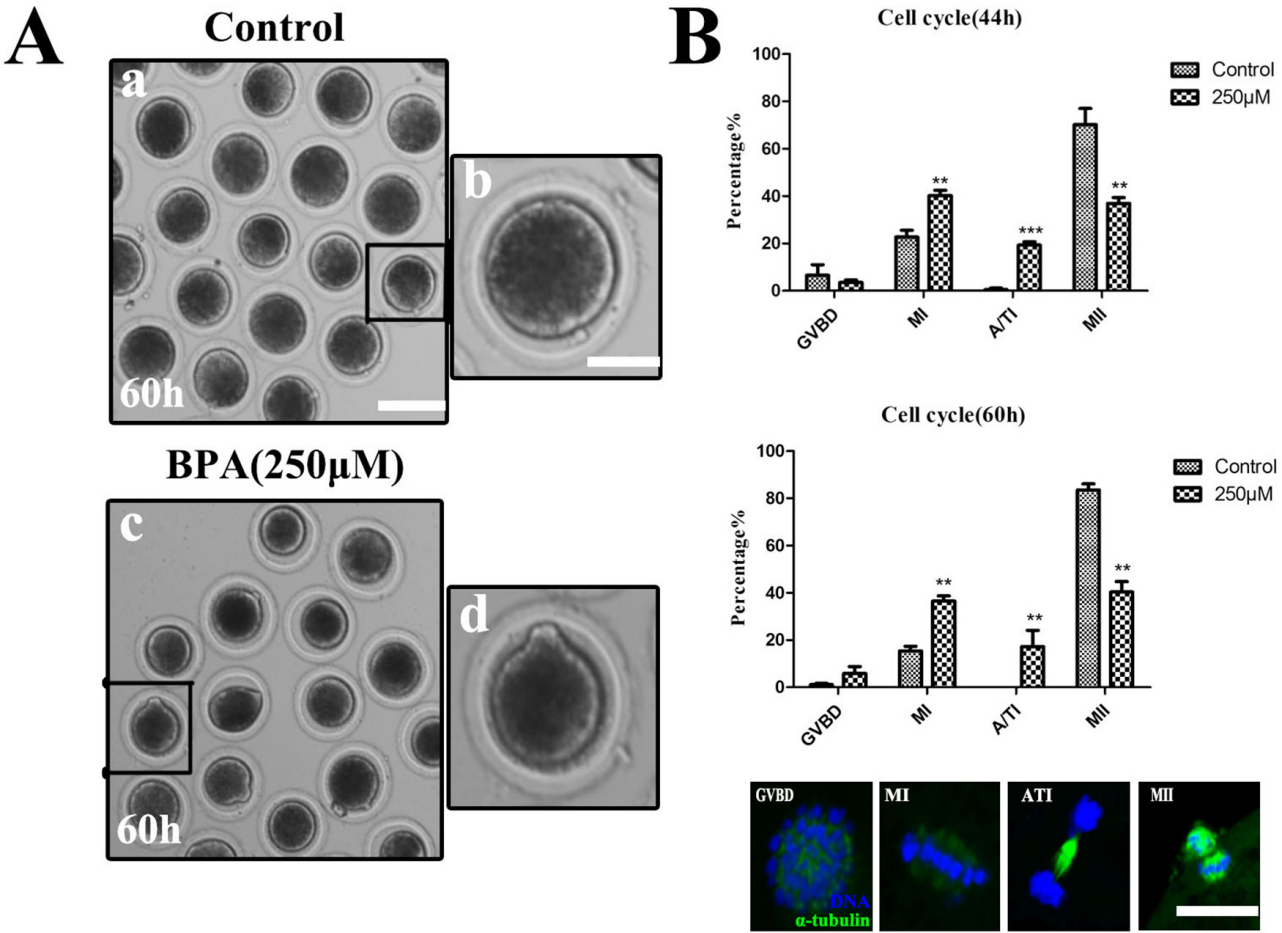
Exposure to BPA disturbs the cell cycle of porcine oocyte maturation **A.** Oocytes failed to extrude polar bodies after exposure to BPA for 60 h culture. a, c Bar = 80 μm; b, d Bar = 20 μm. **B.** The rates of different stages for 44 h and 60 h cultured. (For 44 h, Control n = 114, Treatment n = 151) (For 60 h, Control n = 144, Treatment n = 174), *P < 0.05, the typical stage of porcine oocytes were displayed by the fluorescence images. Green, spindle; blue, chromatin. Bar = 5 μm.

Since BPA treatment caused the failure of oocyte maturation, we extended the culture time to 60 h to check the cell cycle progression. As shown in Figure 2A, we observed the polar body of oocytes for 60 h with BPA culture. Most oocytes had extruded small polar bodies and were arrested at the MII stage in the control group, however most oocytes failed to extrude a polar body with 250 μM BPA treatment. As shown in Figure 2B, COCs were treated with BPA for 44 h, most control oocytes were arrested at meiosis II (MII), whereas most of BPA treated oocytes were arrested at Metaphase I (MI) and anaphase/telophase I (ATI). The proportion of the germinal vesicle breakdown (GVBD) stage oocytes with BPA treatment (3.57 ± 1.00%, n = 151) was not significantly different from that in the control group (6.52 ± 4.53%, n = 114) (P> 0.1). In addition, the rates of MI and ATI stage oocytes were significantly increased (40.18 ± 2.26% for MI and 19.27 ± 1.44% for ATI) compared with controls (22.72 ± 2.78% for MI and 0.57 ± 0.57% for ATI; p<0.01), and the rates of MII stage oocytes were significantly decreased (36.98 ± 2.36%) compared with controls (70.19 ± 6.86%, p<0.05). We also examined the proportion of oocytes stage for 60 h culture, and the result was similar to the above observation. The rates of MI stage were significantly increased to 36.57 ± 2.11% when compared with that in controls (15.34 ± 2.01%) (p<0.01), and similar results was shown at ATI stage (17.17 ± 6.94%, n = 144 vs 0, n = 174); but the rates of MII were significantly reduced (40.41 ± 4.36%) compared with controls (83.54 ± 2.57%) (p<0.01). These results showed that BPA exposure blocked the cell cycle progression of porcine oocyte maturation.

### BPA affects porcine oocyte cytoskeletal dynamics

**Figure 3:**
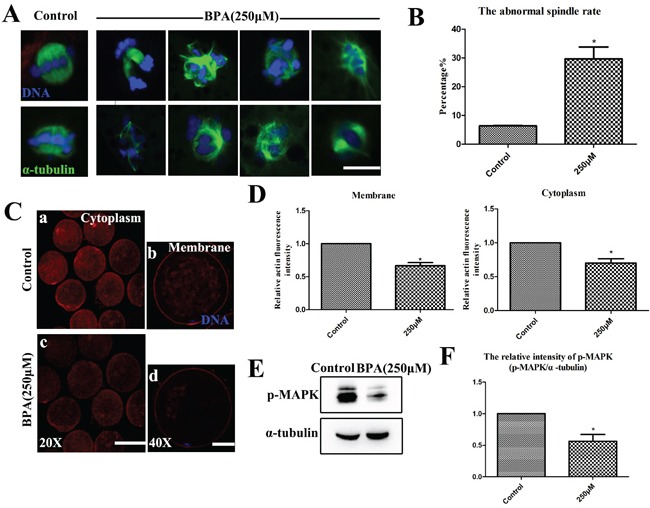
BPA results in oocyte cytoskeletal abnormalities during porcine oocyte maturation **A.** The spindle formation was disrupted after BPA treatment. Bar = 5 μm **B.** The rates of abnormal spindle formation was significantly increased (P < 0.05). **C** and **D.** The expression of actin signals at the membrane and in the cytoplasm of MI oocytes were significantly reduced when compared with controls (P<0.05). a, c Bar = 50 μm; b, d Bar = 20 μm. **E.** The localization and expression level of p-MAPK were assessed by Western blot. **F.** The relative intensity of p-MAPK protein expression (p-MAPK/a-tubulin) was significantly reduced (P < 0.05). Blue, chromatin; Green, tubulin; Red, actin. * P<0.05.

To investigate why the oocytes with BPA treatment reduced maturation competence, We firstly examined the morphologies of meiotic spindles of metaphase I (MI) oocytes after BPA treatment. The majority of control oocytes that were arrested at MI stage had normal spindle morphologies and well-aligned chromosomes. However, the majority of oocytes with BPA treatment had disrupted spindle morphologies and misaligned chromosomes. Spindles showed multiple poles, no poles, or disrupted poles, as shown in Figure 3A. The abnormal rates of oocytes with BPA treatment were significantly increased (29.61 ± 4.17%, n = 110) compared to controls (6.35 ± 0.14%, n = 96, p<0.05), as shown in Figure 3B.

Secondly, we assessed the localization of actin filaments. As shown in Figure 3C, compared with the controls for metaphase I (MI) oocytes after BPA exposure, actin fluorescence intensity on the membrane was significantly reduced (66.55 ± 4.81%, n = 12, vs. 100%; p<0.05). Similarly, the actin fluorescence intensity in the cytoplasm was significantly lower (70.06 ± 6.26%, n = 12, vs. 100%; p<0.05) than that in control MI oocytes (Figure 3D).

To further confirm the effects of BPA on meiotic spindle, we examined the typical spindle regulator MAPK. The oocytes were harvested at MI stage, and then the localization and expression level of p-MAPK were assessed by Western blot. We found that the expression of p-MAPK was decreased after BPA exposure (Figure 3E). For BPA treatment, the level of p-MAPK was significantly lower (56.46 ± 10.6% vs. 100%; p<0.05) compared to controls (Figure 3F).

### BPA treatment results in epigenetic alterations in porcine oocytes

**Figure 4:**
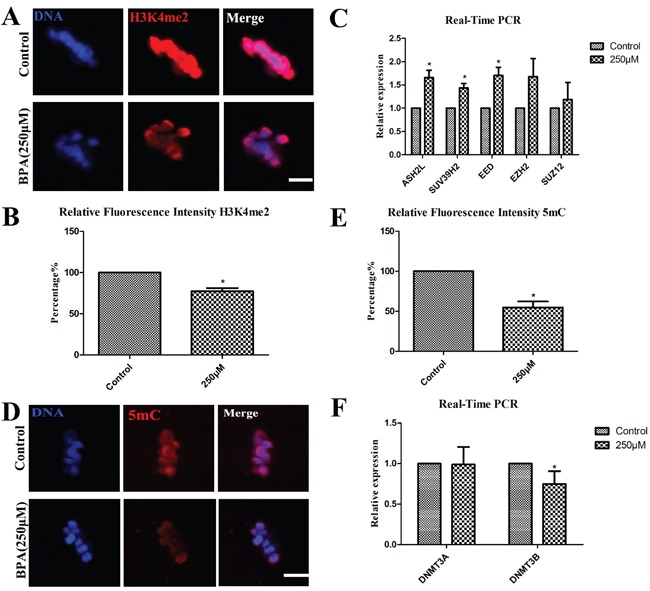
BPA treatment results in epigenetic alterations in porcine oocytes **A** and **B.** The expression of Di-methyl-Histone H3 (Lys4) (H3K4me2) was decreased after BPA treatment by immunofluorescent staining, the fluorescence intensity analysis was significantly decreased (P < 0.05). **C.** *ASH2L, SUV39H2* and *EED* mRNA levels were significantly increased after BPA treatment. **D** and **E.** The expression of 5 methyl cytosine (5 mC) was decreased after exposure to BPA, the fluorescence intensity analysis was significantly decreased (P < 0.05). **F.** *DNMT3B* mRNA levels were significantly decreased after BPA treatment. * P <0.05. Bar =5 μm.

Epigenetic modifications were examined after BPA exposure. Firstly, we harvested the oocytes at the stage of GV and cultured for 26 h to the MI stage, then the oocytes were observed by a confocal microscope after immunofluorescence staining. The levels of histone lysine methylation were examined, and we found that H3K4me2 was co-localized with DNA (Figure 4A); however, compared with controls, the levels of H3K4me2 methylation with BPA treated was significantly reduced (0.77 ± 0.39 vs. 1.0; p< 0.05; Figure 4B). The oocytes were harvested at the stage of MI for 26 h, the levels of mRNA expression for DNA methyl-transferases (*Ash2l, Suv39h2, Eed, Ezh2, Suz12*) were examined by quantitative PCR analysis (Figure 4C). After BPA exposure, the relative mRNA expression of *Ash2l, Eed and Ezh2*, significantly increased compared to the control oocytes (1.66 ± 0.16, 1.44 ± 0.09 and 1.71 ± 0.17 vs. 1.0) (P < 0.05). The relative mRNA expression of *Suz12* and *Suv39h2* were not significantly increased (1.67 ± 0.39 and 1.19 ± 0.37 vs. 1.0) when compared with that in controls (P>0.1).

Secondly, the levels of 5 mC were examined, as shown in Figure 4D, we observed that 5mC was also co-localized with DNA. Compared with the controls for which the relative intensity of 5 mC expression was significantly decreased (54.64 ± 7.70% vs.100%) (P < 0.05) after BPA treatment (Figure 4E). Subsequently, the levels of mRNA expression for DNA methyl-transferases (*Dnmt3a, Dnmt3b*) were assessed by quantitative PCR analysis. The relative mRNA expression of *Dnmt3b* was significantly decreased (0.67 ± 0.09 vs. 1.0) when compared with that in controls (P<0.05). The relative expression of *Dnmt3a* mRNA levels had no significant variation when compared with the controls (0.98 ± 0.22 vs. 1.0) (P>0.1) (Figure 4F).

### BPA treatment increases ROS generation in porcine oocytes

**Figure 5:**
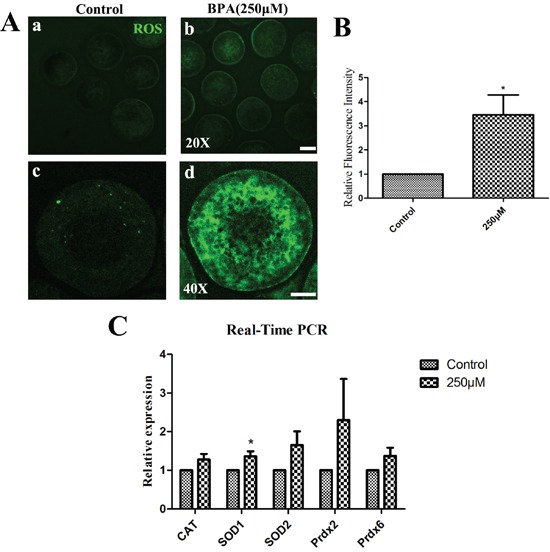
BPA effects on porcine oocyte early apoptosis and autophagy **A.** BPA induced early-stage apoptosis. In control oocytes, it was only show fluorescence on the zona, whereas oocytes exhibited fluorescence on the zona and oocyte membrane after BPA treatment. a, c Bar = 50 μm; b, d Bar = 20 μm. **B.** The early apoptosis rate was significantly increased (P<0.05). **C.** *Bcl-xl* mRNA levels were significantly decreased after BPA treatment. * P <0.05. Bar =20 μm. **D.** Oocytes were stained by immunofluorescence staining after BPA treatment, there are more LC3 puncta than control oocytes. Bar = 20 μm. **E** and **F.** The localization and expression level of LC3 were assessed by Western blot, therelative intensity of LC3 protein expression (LC3/α-tubulin) was significantly increased (P < 0.01). **G.** *LC3, ATG3* and *ATG7* mRNA levels were significantly increased after BPA treatment, but *mTOR* and *Beclin1* mRNA levels were significantly decreased after BPA exposure. * P <0.05.

Next, the levels of ROS were examined after 26 h culture. As shown in Figure 5A, compared with controls the fluorescent intensity of ROS was increased in the treatment group by immunofluorescent staining (Figure 5A). The relative fluorescence intensity of ROS was significantly increased (3.46 ± 0.82 vs.1.0 for control) (P < 0.05) after BPA treatment for 26 h culture (Figure 5B). The levels of mRNA expression for oxidative stress-related genes (*CAT, SOD1, SOD2, Prdx2, Prdx6*) were examined by quantitative PCR analysis (Figure 5C). Only the relative mRNA expression of *SOD1* was significantly increased when compared with the controls (1.36 ± 0.13 vs. 1.0) (P < 0.05). And the relative mRNA expression of *CAT, SOD2, Prdx2* and *Prdx6* had no significantly variation: 1.28 ± 0.15, 1.65 ± 0.36, 2.29 ± 1.08 and 1.37 ± 0.21 vs. 1.0 for controls (P >0.1).

### BPA treatment results in early apoptosis and autophagy in porcine oocytes

**Figure 6:**
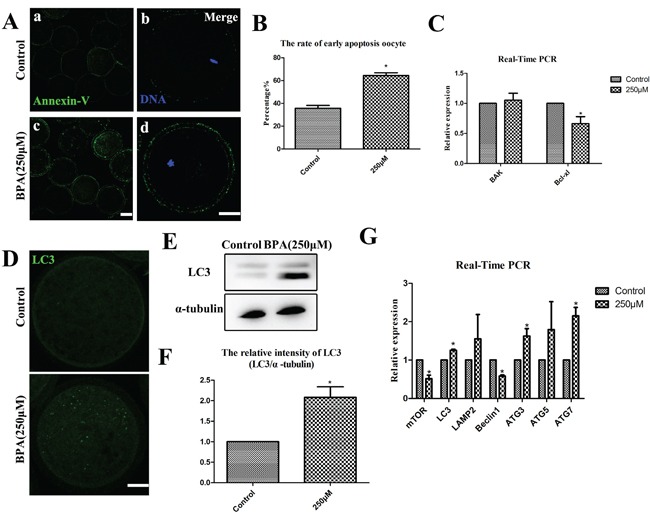
BPA effects on ROS generation after BPA treatment **A.** ROS generation was obviously increased after BPA treatment. a, b Bar = 50 μm; c, d Bar = 20 μm. **B.** The relative fluorescence intensity of ROS was significantly increased (P < 0.05). **C.** The oxidative stress-related genes were examined. *SOD1* mRNA levels were significantly increased after BPA treatment (P < 0.05), while there is no change for the mRNA level of *CAT, SOD2, Prdx2* and *Prdx6*.

Apoptosis and autophagy were examined at the stage of MI after 26 h culture. Firstly, the green fluorescent signals were stained by Annexin-V staining. In control oocytes, Annexin-V staining signals showed at the zona pellucida and were non-apoptotic, but the clear green signals were found in the membrane and zona pellucida after BPA treatment (Figure 6A). As shown in Figure 6B, the green fluorescent signals were significantly increased after BPA treatment (64.31 ± 2.46%, n = 10) compared with controls (35.69 ± 2.46%, n = 10) (P < 0.05). The levels of mRNA expression for apoptosis-related genes (*BAK, Bcl-xl*) were examined by quantitative PCR analysis (Figure 6C). The relative mRNA expression of *Bcl-xl* was significantly decreased when compared to the control oocytes (0.66 ± 0.12 vs. 1.0) (P < 0.05), while the relative expression of *BAK* mRNA levels had no apparent variation when compared with the controls (1.05 ± 0.12 vs. 1.0) (P > 0.1).

Next, as shown in Figure 6D, the status of autophagy were stained by the expression of microtubule-associated protein light chain 3 (LC3), and we found that more dots appeared in the treated oocytes than those in the control oocytes after 26 h culture. Furthermore, the levels of LC3 protein levels were significantly increased after BPA treatment by Western blot (Figure 6E). For control oocytes, the relative intensity of LC3 expression was significantly increased (2.08 ± 0.26 vs.1.0) (P < 0.05) after BPA treatment (Figure 6F). The levels of mRNA expression for autophagy-related genes (*mTOR, LC3, LAMP2, Beclin1, ATG3, ATG5, ATG7*) were examined by quantitative PCR analysis (Figure 6G). The relative mRNA expression of *LC3, ATG3* and *ATG7* were significantly increased when compared to controls (1.26 ± 0.03, 1.63 ± 0.19 and 2.15 ± 0.22 vs.1.0) (P < 0.05). But the relative mRNA expression of *mTOR* and *Beclin1* were significantly decreased when compared to the controls (0.51 ± 0.09 and 0.58 ± 0.03 vs.1.0) (P < 0.05). The results of *LAMP2* and *ATG5* relative mRNA expression had no significant variation: 1.55 ± 0.64 for *LAMP2* and 1.7955 ± 0.73 for *ATG5* vs.1.0 for controls (P > 0.1).

## DISCUSSION

BPA is an endocrine disruptor which could impair human reproductive capacity, and DEHP is an estrogenic chemical that is ubiquitously used in plastics and common consumer products. In this study, our results indicated that exposure to BPA caused failure of polar body extrusion on porcine oocytes *in vitro*, but not DEHP. This may be because that when DEHP enters the gastro-intestinal tract, it is rapidly metabolized to mono(2-ethylhexyl) phthalate (MEHP) and 2-ethylhexanol via pancreatic lipases, while the MEHP has toxic effects instead of DEHP. Subsequently, the toxicity of BPA was demonstrated and its potential mechanisms were explored, including cytoskeletons, epigenetic modification, oxidative stress, autophagy and apoptosis.

Previous study showed that the numbers of porcine COCs which reached complete expansion were significantly reduced after exposure of FSH-stimulated OCC to BPA [30]. BPA exposure suppressed polar body release in mice [31], and exposure to BPA during *in vitro* oocyte maturation could decrease oocyte quality in Bos taurus [32]. In addition, there was a dose-response association of BPA exposure as for altering the maturation of human oocytes *in vitro* [22]. While our results showed that the expansion of cumulus cells was weakened after BPA treatment, and the rates of polar body extrusion were reduced with BPA treatment in porcine oocytes. However, the polar body extrusion rates have no significant variation after DEHP exposure. Our results indicated that similar with other models, BPA had directly toxic effects on porcine oocytes *in vitro*, but not DEHP. In mice, oocytes exposed to high doses of BPA experienced a cell cycle delay, and managed to progress to MII when the culture period was prolonged [31]. And short exposure to BPA during the final stages of oocyte maturation is associated with cell cycle delays and spindle abnormalities [17, 22, 33]. However, our study showed that BPA blocked the cell cycle progression of porcine oocyte maturation even after culturing 60 h. This indicated that porcine oocytes were more sensitive to the BPA exposure.

Actin microfilament and spindle formation are crucial for oocyte meiotic maturation and fertilization. BPA exhibited a significantly higher incidence of meiotic arrest and spindle abnormalities after exposed denuded mouse oocytes *in vitro* [17]. Similar to their findings, our results showed that BPA exposure disrupted spindle formation in porcine oocytes, which also was confirmed by the decreased expression of phosphorylation of MAPK, a regulator which regulated spindle formation [34]. Actin filament is critical for cytokinesis and polar body extrusion, and we also observed reduced the expression of membrane and cytoskeletal actin after BPA treatment. This provided a reason for the failure of polar body extrusion from subcellular structure level.

The epigenetic effects include histone modification, DNA methylation and the formation of histone variants [35]. Previous work showed that BPA might contribute to chromosome congressional failures, and altered gene expression that might affect the health of the offspring [36]. In our results, the decreased level of H3K4me2 indicated that BPA had altered oocyte histone modifications. We also found some epigenetic genes increased after BPA treatment. Because fully grown oocytes are transcriptionally inactive, then the “increases” in mRNA observed might only reflect a failure to degrade the mRNAs. DNA methylation studies shown that BPA exposure significantly changed the methylation levels of differentially methylated regions (DMRs) including the Snrpn imprinting control region (ICR) and Igf2 DMR1 [37]. Thus, the reduced 5 mC expression in our results indicated that BPA had disturbed oocyte DNA methylation status and might further inhibited meiotic progression of oocytes.

Apoptosis also plays a major role in all the stages of oogenesis and even after ovulation [24, 38, 39]. And a previous study showed that implication of autophagy against BPA-mediated neurodegeneration through involvement of AMPK and mTOR pathways [40]. Our results showed that BPA induced early apoptosis and autophagy in porcine oocytes which was confirmed by the levels of LC3 and autophagy-related genes mRNA expression. To further explore this, we also examined the oxidative stress levels. And we found that the porcine oocytes suffered oxidative stress after BPA treatment, showing with increased levels of ROS and oxidative stress-related gene mRNA expression. This result is similar with the previous study which showed that BPA induced oxidative toxicity and carcinogenic effects with a specific dose level [41]. Reactive oxygen species (ROS) play a role in the physiological processes of meiotic resumption as key signal molecules, and including the induction of cell apoptosis and senescence [42]. Therefore, our results indicated that BPA exposure induced ROS, which may further induce apoptosis/autophagy.

In summary, our results showed that the effects of BPA treatment on oocyte cytoskeletons, epigenetic modification, oxidative stress, autophagy and apoptosis of porcine oocytes, which further provided the evidence for the toxic effects of BPA on reproductive systems.

## MATERIALS AND METHODS

### Antibodies and chemicals

BPA and DEHP were purchased from J&K Chemical Ltd. (Shanghai, China). Mouse monoclonal anti-α-tubulin-FITC, phalloidin-TRITC (actin) and Hoechst 33342 were from Sigma (St. Louis, MO, USA). Rabbit polyclonal anti-H3K4me2 antibody, rabbit monoclonal anti-p-MAPK antibody and rabbit polyclonal LC3 antibody were from Cell Signaling Technology (Devers, MA, USA). Mouse monoclonal anti-5-methyl cytosine (5 mC) antibody was purchased from Abcam (Cambridge, UK). Annexin V-FITC/EGFP Apoptosis Detection Kits were purchased from Vazyme Biotech Co., Ltd. (Nanjing, China). Alexa Fluor 488 goat anti-rabbit, 594 goat anti-mouse antibodies were from Invitrogen (Carlsbad, CA, USA).

### BPA and DEHP treatment

BPA and DEHP were dissolved in DMSO respectively, and then BPA was diluted into a final concentration of 200 μM and 250 μM with maturation medium; meanwhile, DEHP was diluted with maturation medium to final concentrations of 250 μM, 500 μM, 750 μM, 1 mM and 5 mM, both of them with the final concentration of the solvent not more than 1% of the culture medium.

### Immunofluorescence staining

Oocytes were fixed with 4% (w/v) paraformaldehyde in phosphate buffered saline (PBS) at room temperature for 30 min which for immunofluorescent staining. Then they were transferred to a membrane permeabilization solution (1% Triton X-100 in PBS) for 8-12 h at room temperature. In blocking buffer (1% bovine serum albumin [BSA] in PBS) for 1 h, oocytes were subsequently incubated overnight at 4°C or 4 h at room temperature with different primary antibodies (H3K4me2, 1:1000; LC3, 1:100). After three washes (2 min each) in washing buffer containing 0.1% Tween 20 and 0.01% Triton X-100 in PBS, oocytes were stained for 1 h with Alexa Fluor secondary antibodies at room temperature. For spindle and actin examination, oocytes were incubated with anti-α-tubulin-FITC and Phalloidin-TRITC staining for 1 h, then samples were counterstained with Hoechst 33342 (10 μg/ml in PBS) for 10 min and then followed by three washes in washing buffer.

Detecting the fluorescence signal of 5 mC, the zona pellucid of oocytes were removed (0.05% pronase in PBS). These oocytes were denatured with 2N HCl at room temperature for 30 min and neutralized with 100 mM Tris-HCl, pH 8.5 for 10 min, then incubated in PBS containing 0.05% Tween-20 at 4°C overnight or at room temperature with for 1 h followed by washing five times in phosphate-buffered saline (PBS) that contained 1% BSA (PB1), then these oocytes were incubated overnight at 4°C with a mouse anti-5-mC antibody (1:500).

Specimens were mounted on glass slides and examined with a confocal laser-scanning microscope (Zeiss LSM 700 META, Germany). In each experiment was repeated at least three times and at least 10 oocytes were examined for each group.

### Oocyte treatment and in vitro maturation

Porcine ovaries were collected from prepubertal gilts at a local slaughterhouse and transported to our laboratory in physiological saline (0.9% NaCl) containing 500 IU/ml of both penicillin and streptomycin at 30-35°C within 2 h kept in a thermos bottle after slaughter. Subsequently ovaries were washed twice with sterile phosphate-buffered saline (PBS), then COCs (cumulus oocyte complexes) were aspirated from antral follicles (2-5 mm in diameter) using a 20-gauge needle attached to a 10-ml disposable syringe. After washing three times with maturation medium, the COCs with intact and compact cumulus were separated from the cellular debris. The medium used for maturation culture was improved TCM-199 supplemented with 75 μg/ml of penicillin, 50 μg/ml of streptomycin, 0.5 μg/ml of LH, 0.5 μg/ml of FSH, 10 ng/ml of epidermal growth factor (mouse EGF; Sigma) and 0.57 mM cysteine (Sigma). To prepare mature oocytes in vitro, a group of 80 COCs were transferred to 500 μl of maturation medium, then medium were subsequently covered with 200 μl paraffin oil in a four-well dish at 38.5°C in a humidified atmosphere of 5% CO^2^ (Nunc, Roskilde, Denmark).

### Annexin-V staining of oocytes

According to the manufacturer's instructions (Beyotime Institute of Biotechnology Hangzhou, China), oocytes were stained with an Annexin-V staining kit. After washing twice in PBS, then viable oocytes were stained for 10 min in the dark with 100 ml of binding buffer containing 5 ml of Annexin-V-FITC. Fluorescent signals were measured by a confocal microscope (Zeiss LSM 700 META).

### Determination of ROS generation

To determine the levels of intracellular ROS production, cumulus-denuded oocytes were incubated with the oxidation-sensitive fluorescent probe [dichlorofluorescein (DCFH)] for 30 min at 37°C in D-PBS that contained 10 μM DCFH diacetate (DCFH-DA) (Beyotime Institute of Biotechnology, China). Then oocytes were washed three times in D-PBS containing 0.1% BSA and then placed on glass slides. The measurement of the intensity of florescence in each oocytes was by a Zeiss LSM 700 META confocal system with the same scanning settings were used for each sample.

### Protein extraction and western blot analysis

A total of 100 porcine oocytes were collected at the MI stage, and then lysed in Laemmli sample buffer (SDS sample buffer with 2-mercaptoethanol), oocytes were subsequently boiled at 100°C for 10 min and immediately transferred to fridge to frozen at −20°C until use. Proteins were separated by SDS polyacrylamide gel electrophoresis (PAGE) using 8% gels and then transferred onto a polyvinylidene fluoride membrane (Millipore, Billerica, MA). Membranes were blocked with Trisbuffered saline (TBS) containing 0.1% (w/w) Tween 20 (TBST) and 5% (w/v) nonfat dry milk powder for 1.5 h at room temperature, and then incubated with a rabbit monoclonal antibody (1:2000; for p-MAPK and LC3, incubation buffer was 5% BSA in PBST) overnight at 4°C. After washing three times in PBST (10 min each), membranes were incubated for 1 h with secondary anti-rabbit HRP-conjugated antibodies (1:2000) in 5% nonfat dry milk in TBST. Finally, the membrane was exposed to enhanced chemiluminescence reagent (EMD Millipore, Billerica, MA).

### Real-time quantitative PCR

30 oocytes were used to extract total RNA with a Dynabead mRNA DIRECT kit (Invitrogen Dynal, Oslo, Norway). First strand cDNA was synthesized using a cDNA synthesis kit (Takara) using Oligo(dT) 12–18 primers (Invitrogen) according to the manufacturer's instructions (Invitrogen). These cDNAs were diluted 10 times and stored at −20°C until analysis. Quantitative real-time PCR (qRT-PCR) was conducted with a fast real-time PCR system (ABI Step One Plus). *GAPDH* was used as a control gene and triplicate samples were assessed for each gene of interest. Relative expression levels were determined by the 2^−ΔΔCt^ method.

### Statistical analysis

For each treatment, at least three biological replicates were done and the results were expressed as means±SEMs. The variance (ANOVA) analysis were used for Statistical comparisons and Duncan's multiple comparisons test were used in differences between BPA treatment groups. The value of p<0.05 was significant.
